# Prevalence and risk factors for brucellosis seropositivity in cattle in Nyagatare District, Eastern Province, Rwanda

**DOI:** 10.4102/jsava.v89i0.1625

**Published:** 2018-12-05

**Authors:** Gervais Ndazigaruye, Borden Mushonga, Erick Kandiwa, Alaster Samkange, Basiamisi E. Segwagwe

**Affiliations:** 1 School of Animal Sciences and Veterinary Medicine, University of Rwanda, Rwanda; 2 Department of Biomedical Sciences, University of Namibia, Namibia; 3 Department of Clinical Studies, University of Namibia, Namibia; 4 Department of Biomedical Sciences, University of Botswana, Botswana

## Abstract

A survey involving 120 small-scale dairy farmers was carried out to assess risk factors associated with brucellosis in cattle from selected sectors of Nyagatare District, Rwanda. A sample of cattle from nine selected sectors of Nyagatare was tested for brucellosis using the Rose Bengal Test. Of the respondents, 57.5% were unaware of brucellosis as a disease, 85.8% did not screen new additions to the herd for brucellosis and 82.5% did not remove brucellosis seropositive animals from the herd. The prevalence of brucellosis in herds with cows with no history of abortion was 38.5% and 17.0% in those with a history of abortion. None of the respondents disinfected abortion sites or vaccinated against brucellosis. The prevalence of brucellosis in cows with a history of retained placenta was 36% and 2% in those with no history of retained placenta. Of the respondents, 62.5% reportedly fed foetal membranes to dogs. About 65.8% of the respondents with brucellosis-positive animals reported a calving interval longer than 1 year. Katabagemu (28.6%) had the highest prevalence of brucellosis seropositivity while Karama had none. Brucellosis in cows (21.4%) was significantly higher than that in heifers (12.8%) (*p* < 0.05), but there was no significant difference between heifers and bulls or between bulls and cows (*p* > 0.05). The occurrence of brucellosis in herds with 40–70 cattle (26.9%) was significantly greater than the 14.9% of herds with 10–39 cattle (*p* < 0.05). Seropositivity to brucellosis in cross-breed cattle (23.6%) was significantly greater than that in indigenous cattle (13.8%) (*p* < 0.05). There was no significant difference in the overall prevalence of brucellosis in cattle from different grazing systems (*p* > 0.05). Seropositivity to brucellosis was significantly different (*p* < 0.05) between the fourth parity (32.5%) and first parity (14.3%) cows. The findings in this study confirmed the existence of brucellosis as a problem in Nyagatare and the authors recommend that farmer education on the epidemiology, risk factors and mitigation of the disease be undertaken as a matter of urgency.

## Introduction

Bovine brucellosis has been eradicated in many developed countries but remains endemic in the developing world as a result of, among other factors, lack of resources and/or control programmes (Akinseye et al. 2016). Brucellosis causes huge economic losses to the farmer through birth of weak calves, stillbirths, prolonged inter-calving intervals, infertility in male animals and interference in the breeding programme of cows that aborted (McDermott, Grace & Zinsstag 2013; Singh, Dhand & Gill 2015; Tasaime et al. 2016). The gold standard for the diagnosis of brucellosis is isolation, culture and identification of the bacterium (Godfroid et al. 2011). However, the turnaround time for these procedures may preclude their use under field conditions. Consequently, various serological tests have been developed to provide rapid acceptable results. Risk factors for brucellosis can be categorised into animal, management and environmental factors. Animal factors include age, breed and sex of the animal; a history of retained placenta and abortion; parity; and milking method (De Alencar Mota et al. 2016; Tasaime et al. 2016; Zeng et al. 2017). Management risk factors for brucellosis include production system (intensive or extensive), screening of new arrivals, hygiene, awareness of the disease, vaccination, herd size and breeding practice (De Alencar Mota et al. 2016; Makita et al. 2011). Environmental factors are mainly associated with agro-ecological location of the animals in either endemic or brucellosis-free locations (Alhaji, Wungak & Bertu 2016; Ogugua et al. 2015).

Risk factors for human brucellosis infection include eating raw meat but predominantly ingesting unpasteurised milk and cheese from infected animals and contact with placentas from aborted foetuses (Alhaji et al. 2016). Control of brucellosis is focused on vaccination, which protects only 60% of the vaccinated animals (Kauffman et al. 2016), eradication, risk factor analysis (Godfroid et al. 2011) and test and slaughter (Caetano et al. 2016). Many countries in Africa have adopted some risk mitigation or management strategies to control brucellosis (McDermott et al. 2013).

There are a number of reports from different countries and, in some cases, same countries giving different estimates of brucellosis prevalence obtained from different serological tests in the East African sub-region (McDermott et al. 2013). In this region, *Brucella abortus* epidemiology appears to be well studied in Uganda, where prevalence rates varying from 6.5% to 44.0% have been reported. One study reported a prevalence of 6.5% using competitive enzyme-linked immunosorbent assay (c-ELISA) for *B. abortus* antibodies (Makita et al. 2011). On the other hand, other researchers reported a prevalence rate of 7.5% using the ELISA confirmed with c-ELISA (Mugizi et al. 2015). A prevalence of 44.0% was reported in Uganda using the Rose Bengal plate test (RBPT) (Nina et al. 2017). In Kenya, a prevalence rate of around 19.0% using the milk ring test (MRT) was reported (Njuguna et al. 2017). In Tanzania, prevalence rates of 48.0% and 21.5% were reported, using indirect enzyme-linked immunosorbent assay (I-ELISA) and RBPT, respectively (Mathew et al. 2015). The bacteriological and serological evidence as well as epidemiological characteristics of brucellosis in sub-Saharan Africa have been recently reviewed (Ducrotoy et al. 2017). There are few publications on bovine brucellosis (Chatikobo et al. 2009; Manishimwe et al. 2015; Rujeni & Mbanzamihigo 2014) and even fewer on the epidemiology of brucellosis in humans (Gafirita et al. 2017; Rujeni & Mbanzamihigo 2014) in Rwanda.

Small-scale dairy production provides a means of livelihood for many farmers in Nyagatare District (Mazimpaka et al. 2017; Mushonga et al. 2017). The prevalence of brucellosis using the Rose Bengal Test (RBT) has been established (Chatikobo et al. 2008). However, these prevalence figures have never been verified by other studies. Furthermore, the animal health, human and socio-economic significance of brucellosis warrant scrutiny. Identification of risk factors associated with brucellosis is important in devising an effective control strategy for the disease (Kumar, Bharathi & Porteen 2017). To the best of our knowledge, the risk factors for brucellosis for cattle in Nyagatare and Rwanda at large have never been analysed. The aim of this study was to analyse the risk factors associated with brucellosis in cattle from selected sectors of Nyagatare District, Rwanda.

## Materials and methods

### Study area

Nyagatare District is one of seven districts of the Eastern Province of Rwanda, forming a border with Uganda to the north and Tanzania to the east. The district consists of about 1743 km^2^ of land and is divided into 14 sectors with 16 milk collection centres (Karushuga, Mbare, Rwabiharamba, Musenyi, Kamate, Katabagemu, Abashumbabeza, Nyagatare, Rwempasha, Tabagwe and Karama) and one milk processing plant Inyange. Collected milk is destined for immediate sale in Kigali or for processing and eventual sale in Nyagatare. Nyagatare residents survive mainly on crop and livestock production. The district is home to more than 106 822 cattle that consist predominantly of the Ankole breed (60 818 cattle) and various cross-breeds (46 004 cattle). Although there is a tendency towards fencing these small-scale farms (Mazimpaka et al. 2017), most mixed cattle herds share grazing and breeding is uncontrolled.

### Study design and sampling procedure

A stratified random sampling design was used to select farmers to answer a close-ended questionnaire (Table 1) with the assistance of pretrained agricultural extension personnel with a good command of the local *ikinyarwanda* language under the supervision of the authors. Farmers were randomly chosen for the survey by picking names out of a hat. Farmers known to supply milk to the milk centres formed the strata in this design. A tenth of the *Brucella* unvaccinated cattle belonging to the respondents were randomly selected, by picking their identities out of a hat, for determination of the serostatus for brucellosis.

**TABLE 1:** The proportional responses of respondents (*n* = 120) to questions addressing management-related risk factors.

Risk factors	Positive responses		Negative responses	
	Number	% (proportion)	Number	% (proportion)
Aware of brucellosis as a disease	51	42.5	69	57.5
Screening of new animals	17	14.2	103	85.8
Removal of disease seropositive animals	21	17.5	99	82.5
Previous history of abortion(s) in herd	109	90.8	11	9.2
Previous history of retained placenta(s) in herd	50	41.7	70	58.3
Calving interval > 1 year	79	65.8	41	34.2
Fed foetal membranes to dogs	75	62.5	45	37.5
Disinfected abortion site(s)	0	0.0	120	100.0
Vaccinated against brucellosis	0	0.0	120	100.0

### Rose Bengal Test

Blood was collected from the sampled animals by sterile coccygeal venipuncture in plain (red top) vacutainer blood tubes. After clotting, tubes were centrifuged at 5000 revolutions per minute for 5 min and the harvested sera were frozen at -30 °C in properly labelled cryovials until the time of assaying. The RBT kit (Sigma-Aldrich, St Louis, USA) was used according to the World Organisation for Animal Health (Office International des Epizooties [OIE]) guidelines (OIE 2016). Non-haemolysed blood (clear) sera and control sera were thawed to room temperature 30 min before use.

### Statistical analysis

The survey and RBT results were summarised in Excel 2013 and then imported to the Statistical Package for Social Sciences version 16.0 for analysis using Pearson’s chi-square test and the *Z* test for comparison of proportion: *p* < 0.05 was considered significant. Odds ratios and *p*-values were calculated for seropositivity to brucellosis between parity, sex, reproductive and herd size categories.

## Results

The results in Table 1 showed 57.5% of respondents were unaware of brucellosis as a disease. The majority (85.8%) of the respondents did not screen new additions to the herd for brucellosis. Many (82.5%) of the respondents did not remove *Brucella*-positive animals from the herd. Most of the respondents (90.8%) experienced abortions in their herds. About 58.3% of the respondents, however, did not experience retained placentas in their cows. A significantly larger proportion of the respondents reported a calving interval longer than one year (*p* < 0.05). A significantly larger proportion of the respondents reportedly fed foetal membranes to dogs (*p* < 0.05). None of the respondents disinfected abortion sites or vaccinated against brucellosis.

The prevalence of seropositivity to bovine brucellosis in the different groups of animals in the different districts is presented in Table 2. The results in Table 2 show that Katabagemu (28.6%) had the highest overall prevalence of brucellosis followed by Tabagwe (26.9%), Nyagatare (23.1%), Rukomo (21.4%), Rwempasha (17.6%), Rwimiyaga (17.3%), Gatunda (14.3%), Karangazi (5.6%) and Karama (0%). Overall, statistical analysis of the results in Table 2 using the chi-square test for independence showed that there was no significant difference in the prevalence of brucellosis among the different sectors (*p* > 0.05). However, the individual cross-tabulation of the overall prevalence of brucellosis between sectors using the *Z* test revealed that the prevalence of brucellosis in Karangazi (5.6%) and Karama (0%) was significantly lower than in the rest of the sectors (*p* < 0.05). There was no significant difference in the prevalence of brucellosis between Karangazi and Karama sectors (*p* > 0.05).

**TABLE 2:** Prevalence of brucellosis in cattle in Nyagatare District.

Sector	Positive cases		Negative cases		Total number of cases
	Number	% (proportion)	Number	% (proportion)	
**Katabagemu**					
Cows	10	29.4	24	70.6	34
Bulls	2	25	6	75	8
Heifers	0	0	0	0	0
Overall	12	28.6	30	71.4	42^a^
**Nyagatare**					
Cows	20	27.8	52	72.2	72
Bulls	0	0	0	0	0
Heifers	4	12.5	28	87.5	32
Overall	24	23.1	80	76.9	104^a^
**Tabagwe**					
Cows	12	27.3	32	72.7	44
Bulls	0	0	0	0	0
Heifers	2	25	6	75	8
Overall	14	26.9	38	73.1	52^a^
**Rukomo**					
Cows	6	30	14	70	20
Bulls	0	0	0	0	0
Heifers	0	0	8	100	8
Overall	6	21.4	22	78.6	28^a,b^
**Rwempasha**					
Cows	10	15.2	56	84.8	66
Bulls	0	0	10	100	10
Heifers	8	30.8	18	69.2	26
Overall	18	17.6	84	82.4	102^a,b^
**Rwimiyaga**					
Cows	26	21.7	94	78.3	120
Bulls	4	22.2	14	77.8	18
Heifers	6	8.6	64	91.4	70
Overall	36	17.3	172	82.7	208^a,b^
**Gatunda**					
Cows	2	25	6	75	8
Bulls	0	0	0	0	0
Heifers	0	0	6	100	6
Overall	2	14.3	12	85.7	14^a,b,c^
**Karangazi**					
Cows	2	5.6	34	94.4	36
Bulls	0	0	0	0	0
Heifers	0	0	0	0	0
Overall	2	5.6	34	94.4	36^b,c^
**Karama**					
Cows	0	0	12	100	12
Bulls	0	0	0	0	0
Heifers	0	0	6	100	6
Overall	0	0	18	100	18^c^

**Overall**	**114**	**18.9**	**490**	**81.1**	**604**

a,b,c , Sectors sharing the same superscript were not significantly different (*p* > 0.05); districts with different superscripts were significantly different (*p* < 0.05).

The prevalence of brucellosis in cross-breed cattle was 23.6%, and in indigenous cattle it was 13.8%. The prevalence of brucellosis in male animals was 16.7% in comparison to 19% in female animals. The prevalence of brucellosis in extensively grazed cattle was 19.1% in comparison to 0.0% in intensively grazed cattle. The proportion of *Brucella*-negative female animals (81.0%) was significantly greater than that of *Brucella*-positive female animals (19%). Cows in their fourth parity had the highest prevalence of brucellosis (32.5%) followed by those in their third parity (27.7%), second parity (18.6%), fifth parity (16.7%) and first parity (14.3%). Table 4 also shows that more (21.4%) cows were positive for brucellosis than bulls (16.7%) and heifers (12.8%). The prevalence of brucellosis was greater in cows from herds with no history of abortion (38.5%) than from herds with a history of abortion (17.0%). The table also shows that the prevalence of brucellosis in cows with a history of retained placenta was 36.0% and in cows with no history of retained placenta was 2.0%.

**TABLE 3:** Prevalence of brucellosis according to animal-related risk factors.

	Brucellosis-positive		Brucellosis-negative		*p*
	Number	% (Proportion)	Number	% (Proportion)	
**Breed**	-	-	-	-	0.001
Indigenous	40	13.8	250	86.2	-
Cross-breed	74	23.6	240	76.4	-
**Sex**	-	-	-	-	0.460
Female animals	108	19.0	460	81.0	-
Male animals	6	16.7	30	83.3	-
**Abortion**	-	-	-	-	0.000
History of abortion	94	17.0	458	83.0	-
No history of abortion	20	38.5	32	61.5	-
**Retained placenta**	-	-	-	-	1.470
History of retained placenta	108	36.0	192	64.0	-
No history of retained placenta	6	2.0	298	98.0	-
**Parity**	-	-	-	-
0 (heifers)	20	12.8	136	87.2	^a^
1st	14	14.3	86	87.8	^a^
2nd	18	18.6	77	79.4	^a^
3rd	18	27.7	47	72.3	^a^
4th	26	32.5	54	67.5	^a^
5th	12	16.7	60	83.3	^a^
Overall	108	19.0	460	81.0	-
**Reproductive category**					
Cows	88	21.4	324	78.6	^a^
Bulls	6	16.7	30	83.3	^a^
Heifers	20	12.8	136	87.2	^a^
Overall	114	18.9	490	81.1	^a^
**Grazing system**	-	-	-	-	0.28
Extensive grazing	114	19.1	484	80.9	-
Intensive grazing	0	0.0	6	100.0	
**Herd size**					
1–9 cattle	0	0.0	6	100.0	^a^
10–39 cattle	58	14.9	332	85.1	^a^
40–70 cattle	56	26.9	152	73.1	^a^

Confidence intervals and *p*-values are calculated in Table 4.

a , Odds ratios.

**TABLE 4:** Odds ratios, confidence intervals and *p*-values of different categories of cattle.

Compared categories	Odds ratio (95% CI)	*p*
1st parity cows versus heifers	1.11 (0.53–2.31)	0.46
2nd parity cows versus 1st parity cows	1.44 (0.67–3.08)	0.23
2nd parity cows versus 5th parity cows	1.17 (0.52–2.61)	0.43
4th parity cows versus 3rd parity cows	1.26 (0.61–2.58)	0.33
3rd parity cows versus 5th parity cows	1.91 (0.84–4.37)	0.09
5th parity cows versus 1st parity cows	1.14 (0.49–2.65)	0.46
3rd parity cows versus 2nd parity cows	1.64 (0.78–3.46)	0.13
4th parity cows versus 1st parity cows	2.96 (1.42–6.16)	0.00^a^
Bulls versus heifers	1.36 (0.50–3.68)	0.35
Cows versus bulls	1.36 (0.55–3.37)	0.34
Cows versus heifers	1.85 (1.09–3.12)	0.01^a^
Female versus male animals	1.17 (0.48–2.89)	0.46
10–39 cattle herd versus 1–9 cattle herd	∞	0.16
40–70 cattle herd versus 1–9 cattle herd	∞	0.38
40–70 cattle herd versus 10–39 cattle herd	2.11 (1.09–3.19)	0.00^a^

CI, confidence interval.

a , Significant difference between categories.

Table 5 shows the odds ratios, confidence intervals (CIs) and *p*-values of the cross-tabulations of prevalence of brucellosis between parities, reproductive categories, male and female animals and herd size. Herds with 40–70 cattle showed 26.9% seropositivity in comparison to those with 10–39 cattle (14.9%) and seropositivity in cows in the fourth parity (32.5%) was significantly higher than in cows in the first parity (14.3%) (*p* < 0.05). Significantly more cows (21.4%) than heifers (12.8%) were seropositive (*p* < 0.05).

**TABLE 5:** Odds ratios of categories with statistically insignificant differences since *p* > 0.05.

Compared categories	Odds ratio
1st parity cows versus heifers	1.107
2nd parity cows versus 1st parity cows	1.436
2nd parity cows versus 5th parity cows	1.169
4th parity cows versus 3rd parity cows	1.257
3rd parity cows versus 5th parity cows	1.915
Bulls versus heifers	1.36
Cows versus bulls	1.358
Female versus male animals	1.174

## Discussion

The results obtained in this investigation confirm the endemic nature of bovine brucellosis in the Nyagatare District. A high prevalence of brucellosis in cattle occurs when more than 35% of the herds and more than 10% of individual animals are infected (WHO 1965). It is difficult to determine whether there was a high prevalence in this study as the herd prevalence is below the cut-off point of 35% while the individual prevalence is more than the cut-off point of 10%. Previous investigations carried out in milking cows in Nyagatare District using the RBT have revealed a bovine brucellosis prevalence of 10% (Chatikobo et al. 2008). Results from this study therefore show that the prevalence of brucellosis in this district has nearly doubled in 10 years. These results are a cause for concern for medical and veterinary practitioners in Nyagatare District, and indeed, in the whole of Rwanda. Recent studies in Huye District also confirmed 25% seroprevalence of brucellosis in women and 7.4% seroprevalence in cattle (Rujeni & Mbanzamihigo 2014). Yet another study has confirmed seroprevalence of bovine brucellosis of 2.3% in Kigali City (Manishimwe et al. 2015). A very recent study at Nyagatare Hospital reported 6.1% seroprevalence of brucellosis in humans (Gafirita et al. 2017), confirming that the disease is endemic in the area. In order to effectively control brucellosis, there is an urgent need to develop and implement eradication programmes in view of the risk factors for this disease identified in this study.

Culture and isolation of *Brucella* species from animal samples in selective media (Farrell or modified Thayer-Martin) are recommended by the OIE as the ‘gold standard’ for diagnosis of *Brucella* with 46.1% sensitivity and 100% specificity. Of the conventional tests, the buffered antigen plate agglutination test with 95.4% sensitivity and 97.7% specificity was rated the most accurate by Gall and Nielsen (2004). The RBT (81.2% sensitivity and 86.3% specificity) in this study was chosen for its relatively acceptable accuracy and lower cost (Gall & Nielsen 2004). Various serological screening tests such as buffered acidified antigen, RBPT, serum agglutination test, c-ELISA (Mangi et al. 2015; Tasaime et al. 2016), I-ELISA (Ndwandwe 2015), 2-Mercaptoethanol test or MRT (Bakhtullah et al. 2014), brucellin skin test (Manishimwe et al. 2015) and real-time polymerase chain reaction (Gwida et al. 2016) are currently not readily available in Rwanda. Under normal circumstances, the RBT or MRT on blood and milk, respectively, are used for screening; confirmation is obtained by the complement fixation test (Tadesse 2016) or c-ELISA (Ogugua et al. 2015). Other researchers have pointed out that serological tests alone tend to underestimate the true prevalence of brucellosis and thus results of culture are always higher than those obtained from serology (Júnior et al. 2017).

The RBT is a highly sensitive test for the detection of immunoglobulins IgG1, IgM and possibly IgG2 against *Brucella* in the sera of cattle (Cadmus, Adesokan & Stack 2008). Because of the high sensitivity of the test, false-positive results may be obtained and therefore the RBT is only used as a screening test. Positive test results are confirmed with an additional test such as the serum agglutination test. False positives, limiting the reliability of the RBT, arise from other gram-negative bacteria such as some species of *Vibrio, Escherichia, Yersinia, Stenotrophomonas* and *Salmonella* antigenic determinants with the *Brucella* O-chain (Mainar-Jaime et al. 2005). In addition, false-negative results, though very rare, may occur because of excessive heating of sera above 45 ºC during storage or transit. Furthermore, low post-infection antibody levels could result in false-negative results. If the animals have been vaccinated beforehand, this could result in false-positive RBT results. Antibodies elicited by vaccination of cattle with S19 *Brucella* strain have been recently demonstrated to persist for at least 4.5 years (Simpson et al. 2018). In heifers older than the recommended eight months of age, vaccine-induced antibodies could result in false-positive results. Vaccination is, however, not practised by poor rural farmers in Rwanda.

The results of this study show that a history of retained placenta and a calving interval of greater than 1 year (Table 1); age, parity and breed of animal (Table 3); and reproductive category of animal (Table 4) were the major animal-related risk factors for brucellosis. The results further show that there was a higher prevalence of brucellosis in animals older than 3 years than in animals younger than 3 years, suggesting that older animals have been exposed earlier on and are probably immune and perhaps persistent carriers. There were also more positive cases among cross-breeds (22.7%) than local breeds (13.8%) and more positive cases in herds with no history of abortion and in cases with a history of retained placenta than in herds without such a history. The higher prevalence of brucellosis antibodies in herds with no history of abortion than in those with a history of abortion observed in this study begs an explanation because in most cases, the converse is true. Studies from elsewhere have identified history of abortion as a risk factor for brucellosis (Alhaji et al. 2016; Tasaime et al. 2016) while our results are different. These results may be a random outlier or it is likely that there was another undiagnosed cause of abortions in the herds in this study.

This study was not designed to investigate the breed differences in susceptibility to brucellosis. However, a difference in the seroprevalence of brucellosis in Barka and Arado breeds was observed in Ethiopia (Mekonnen, Kalayou & Kyule 2010). That study further attributed this breed susceptibility to breed management systems as opposed to the breeds per se (Mekonnen et al. 2010). However, it is worth noting that the Ankole (Inyambo) is the dominant breed in Rwanda because of its alleged hardiness and resistance to endemic diseases. It currently makes up 76.0% of the Rwanda herd (Mazimpaka et al. 2017). Several studies have suggested that indigenous breeds are more resistant to brucellosis than exotic breeds (Kumar et al. 2017; Mangi et al. 2015; Matope et al. 2011).

The results of this study on animal-related risk factors for brucellosis are in agreement with several other studies. For instance, the history of retained placentas and abortions has been confirmed by some authors as increasing the odds of brucellosis in their studies (Samaha et al. 2009; Tasaime et al. 2016). In addition, previous studies in other regions of the world reported a higher brucellosis prevalence in older animals (Al Hassan et al. 2014). A higher prevalence of brucellosis in post-pubertal than in prepubertal animals was also reported (Tadesse 2016). According to several authors, the prevalence of brucellosis is higher in female animals than in male animals (De Alencar Mota et al. 2016; Mangi et al. 2015). However, there is one study that reported a higher prevalence in male animals than in female animals (Mai et al. 2012). Our study revealed no significant difference in the prevalence of brucellosis between male and female animals. However, the female animals were 1.174 times more likely to be positive (odds ratio 1.174) for brucellosis in our study. Furthermore, it has been previously suggested that higher parity female animals showed a higher prevalence of brucellosis than lower parity herd mates (Zeng et al. 2017).

Management-related risk factors for brucellosis in this study included awareness of brucellosis by the farmer, screening of new arrivals, removal of reactors, feeding of foetal membranes to dogs, disinfection of abortion sites, brucellosis vaccination (Table 2), grazing system and herd size (Table 4). It is alarming that these results show that 98.0% of the respondents practise extensive grazing and most of the farms with herd size of 39–70 cattle had higher prevalences than those with 1–10 cattle. Only 42.5% of the respondents knew about brucellosis, 14.2% screened new arrivals, 17.5% removed reactors from the herd, 62.5% fed foetal membranes to dogs, none disinfected abortion sites and none vaccinated their cattle against brucellosis. Although it was not statistically significant, more positive cases occurred in herds where extensive grazing was practised (19.0%) than where zero and intensive grazing was used (0.0%).

In contrast to our findings, another study reported a tendency towards intensive grazing in fenced farms, which was the main rearing system in all sectors of Nyagatare, with the exception of Rukomo sector where 50% of the respondents practised zero grazing (Mazimpaka et al. 2017). However, even when livestock is kept on fenced farms, cattle are still herded daily to the sources of water, as most fenced farms do not have water sources (Mazimpaka et al. 2017). It has been argued that extensive and pastoral production systems have a higher risk for brucellosis than intensive and, in particular, stall-fed systems (De Alencar Mota et al. 2016; Makita et al. 2011). According to some authors, pastoralism is positively associated with brucellosis (Boukary et al. 2013; Tadesse 2016).

Previous studies have demonstrated that large herd size is a risk factor for brucellosis (Makita et al. 2011; Tasaime et al. 2016). Another study concluded that the larger the herd, the higher the risk for brucellosis (Megersa et al. 2011). Large herd size provides more opportunities for infection, especially following abortions, through increased contact between the animals and common feeding and watering points promoting transmission of *Brucella* organisms. Larger herd sizes have been found to be more frequently in the Karangazi and Rwemiyaga sectors, with average herd sizes of 70 and 68 head of cattle, respectively (Mazimpaka et al. 2017). Interestingly, Karangazi and Rwemiyaga adjoin game parks and there is a risk of spreading the disease to wild animals in the game parks. It has also been shown that brucellosis was more prevalent on farms where the method of breeding was unknown, followed by the use of community bulls and artificial insemination, in that order (Bakhtullah et al. 2014; Ebrahim et al. 2016). Anecdotal evidence from our study shows that almost all the farmers believe that brucellosis is transmitted by bulls and it was observed that most of the farmers in the study area used natural service. According to two studies, bull exchange for mating and introduction of new animals to the herd are major risk factors for brucellosis (Alhaji et al. 2016; Berhe, Belihu & Asfaw 2007).

Environmental or agro-ecological location factors investigated in this study were mainly based on sectors rather than any agro-ecological regions. In fact, the whole of Nyagatare is a single agro-ecological region. However, Karangazi sector borders the Akagera National Park. Its proximity to the national park has the potential to spread the disease into wildlife reservoirs, making brucellosis even more difficult to control. In fact, since 2005, there has been a process of degazetting parts of Akagera National Park to create more livestock grazing areas (Mazimpaka et al. 2017). This creates an opportunity for livestock and wildlife to co-exist within this grazing area. One study reported that the seroprevalence of brucellosis was higher in wild ruminants where a wildlife–domestic animal interface existed as opposed to where it did not exist (Motsi et al. 2013). The low prevalence of brucellosis in Karangazi works to the advantage of Nyagatare District as risk of spread to the Akagera National Park is lower. However, the occurrence of brucellosis complicates the epidemiology of brucellosis within a given ecosystem. For instance, wildlife was blamed for the re-emergence of brucellosis in livestock within the Greater Yellowstone Area of the United States, following decades of freedom-from-brucellosis status (Olsen 2010).

It would appear that all sectors in Nyagatare District are contiguous with each other in terms of animal movement, as animal movement control in Rwanda is only enforced at provincial level. For the same reason, it is impossible to establish endemic and free zones within the same district. Previous research has reported that there is a higher probability of an animal from a brucellosis-endemic zone testing positive than an animal from brucellosis-free geographical locations (Alhaji et al. 2016; Ogugua et al. 2015).

The higher seroprevalence in Katabagemu sector could be because of large herd sizes and extensive grazing systems practised in the study area. However, being the most populous sector puts more people at risk of contracting the disease. The higher prevalence in both Katabagemu and Rukomo sectors could be explained by their rural location (Boukary et al. 2013). However, the situation in Nyagatare is paradoxical, as the sector is largely urban (capital of Nyagatare District). Using that reasoning, we would expect a lower prevalence in an urban sector with a considerable peri-urban component like Nyagatare sector.

This study did not assess risk factors for humans; however, many risk factors studied for cattle, such as contact with infected afterbirths, not disinfecting abortion sites, non-vaccination of animals, not screening new animals and not eradicating positive reactors, impact directly on humans as they do on cattle. Other behavioural risk factors for brucellosis infection in man include ingesting raw meat and unpasteurised milk from infected animals. Surprisingly, contact with milk droplets and drinking of raw milk from infected cows do not appear to be risk factors for brucellosis in the nomadic Fulani herds of Nigeria (Alhaji et al. 2016). The observation differs from the report in another study, which reported that consumption of unpasteurised milk is a risk factor in humans for brucellosis infection (Rock et al. 2016).

## Conclusion

This study has led to a better understanding of the risk factors associated with bovine brucellosis in livestock in the Nyagatare District. The major risk factors were a history of retained placenta, a calving interval of greater than one year, age of animal, parity of animal, breed and reproductive category of animal. Furthermore, farmers have limited knowledge of bovine brucellosis. Consequently, management does not include practices such as routine screening of replacement stock and vaccinations. The extensive grazing of livestock adjacent to game parks also poses an increased risk of the spread of brucellosis to wildlife. Similarly, the prevalence of human brucellosis in the general population (except in women at hospitals) of Nyagatare District and Rwanda in general is not known and also needs to be investigated.

## Recommendations

Education of farmers on bovine brucellosis and other zoonotic diseases known to be endemic in the region is advocated. In order for Rwanda to reach her objective of attaining self-sufficiency in livestock production, as detailed in the Strategic Plan for the Transformation of Agriculture in Rwanda (MINAGRI 2009), a brucellosis eradication and control programme must be implemented.

Mass vaccination of all animals should be implemented immediately and continued, and subsequent vaccination restricted to replacement stock only. However, this latter option, together with test and slaughter in adult animals, may be unrealistic under extensive pastoralist production systems in sub-Saharan Africa (Blasco & Molina-Flores 2011).

Further research on the epidemiology of brucellosis in wildlife in Nyagatare should be conducted. Research has shown that control of brucellosis in wildlife is extremely difficult if not impossible (Olsen 2010; Treanor 2013). Any programme aiming to control brucellosis in an ecosystem should therefore be targeted at livestock (Ducrotoy et al. 2017; Olsen 2010).
